# The absence of both RIBC1 and RIBC2 induces decreased sperm motility and litter size in male mice

**DOI:** 10.1111/andr.70045

**Published:** 2025-04-23

**Authors:** Kento Katsuma, Keisuke Shimada, Shingo Tonai, Daisuke Mashiko, Rie Iida‐Norita, Yuki Kaneda, Haruhiko Miyata, Masahito Ikawa

**Affiliations:** ^1^ The Institute of Medical Science The University of Tokyo Tokyo Japan; ^2^ Research Institute for Microbial Diseases Osaka University Suita Osaka Japan; ^3^ Immunology Frontier Research Center Osaka University Suita Osaka Japan; ^4^ Graduate School of Pharmaceutical Sciences Osaka University Suita Osaka Japan; ^5^ Center for Infectious Disease Education and Research Osaka University Suita Osaka Japan; ^6^ Present address: Laboratory of Disease Models, School of Veterinary Medicine Rakuno Gakuen University Ebetsu Japan

**Keywords:** doublet microtubules, microtubule inner proteins, RIBC1, RIBC2, sperm motility

## Abstract

**Background:**

RIBC1 (RIB43A domain with coiled‐coils 1) and RIBC2 (RIB43A domain with coiled‐coils 2) are homolog proteins of RIB43a which is localized to microtubules in the cilia and flagella of unicellular organisms. Cryo‐electron microscopy and artificial intelligence studies showed that RIBC1 and RIBC2 are microtubule inner proteins (MIPs) localized in the inner lumen of the doublet microtubules (DMTs) in mouse sperm flagella. However, the function of RIBC1 and RIBC2 in mammalian reproduction and sperm flagella is still unknown.

**Objective:**

To clarify the function of RIBC1 and RIBC2 in mouse spermatozoa.

**Materials and methods:**

We generated *Ribc1* knockout (KO), *Ribc2* KO, and *Ribc1* and *Ribc2* double‐knockout (*Ribc1/2* DKO) mice using the CRISPR/Cas9 system and analyzed their phenotypes.

**Results:**

We revealed that the loss of either RIBC1 or RIBC2 alone did not affect male fertility, but the absence of both caused a decrease in pup numbers. Sperm motility analysis showed that *Ribc1* KO spermatozoa had reduced velocity, but *Ribc2* KO sperm velocities were comparable with WT mice. However, *Ribc1/2* DKO sperm velocities were significantly lower than those from *Ribc1* KO mice. No structural abnormalities in the axonemal structure at the transmission electron microscope (TEM) level and no abnormalities in the flagellar waveform pattern were observed in *Ribc1/2* DKO spermatozoa.

**Discussion and conclusion:**

Both RIBC1 and RIBC2 are not significant for maintaining the axonemal structure in mouse spermatozoa, but both proteins function cooperatively in sperm motility. This result may indicate that minor structural changes due to RIBC protein absence are not detected at the TEM level, and RIBC2 function depends on RIBC1 in sperm motility. We think that reduced litter size in *Ribc1/2* DKO mice is caused by reduced sperm motility due to minor structural abnormalities caused by the loss of two RIBC proteins.

## 1 INTRODUCTION

Spermatozoon consists of a head and a flagellum. The head contains a nucleus and an acrosome, which contain enzymes and functional molecules required for fertilization.^1^, ^2^, ^3^ The flagella comprise a midpiece, principal piece, and endpiece, with all having a common motor apparatus called the axoneme.^4^, ^5^, ^6^ The structure of axoneme is evolutionarily conserved from unicellular organisms to humans^7^. The axonemes of sperm flagella extend early in spermiogenesis (step 2–3 round spermatids in mice) from the distal centriole, and eventually form the central structure throughout the length of the sperm tail.^8^, ^9^ Axonemes share a “9+2” structure consisting of two central microtubules and nine doublet microtubules (DMTs) in the midpiece and principal piece (Figure S1A).^4^, ^5^ DMT of axonemes comprises 13 complete protofilament (PF) A microtubules (A tubules) and 10 incomplete PF B microtubules (B tubules). The luminal side of DMTs is lined with microtubule inner proteins (MIPs).^10^, ^11^, ^12^ Several papers reported that MIPs are important for DMT stability.^10^, ^11^, ^12^ Sperm motility is driven by the coordinated sliding motion of DMTs, in which dynein motors attached to the DMTs use the energy of ATP hydrolysis.^13^, ^14^ In addition, sperm motility enables spermatozoa to migrate through the uterus and oviduct as well as to pass through the zona pellucida, the extracellular matrix surrounding the oocyte, to reach the oocytes.^5^ Therefore, disruption of DMTs caused by the absence of MIPs such as MNS1 and TEKTIP1 induces male infertility or subfertility via abnormal sperm motility.^15^, ^16^

A filamentous MIP, RIB43a, is predicted to be crucial for stabilizing PF ribbons in *Chlamydomonas* and *Tetrahymena*.^17^, ^18^ In *Chlamydomonas*, a unicellular organism, RIB43a is localized to the PF ribbons of the doublet and triplet microtubules of the flagella.^17^ In another unicellular organism, *Tetrahymena*, RIB43a is an MIP that forms the PF ribbons inside the DMTs of the cilia.^18^, ^19^ Molecular dynamics simulations revealed that Rib43a stabilizes the tubulin lattice at the molecular level.^18^ Nevertheless, it is unclear whether the loss of RIB43a will affect the structure of microtubules. There are no RIB43a in vertebrates, rather, they have RIBC1 (RIB43A domain with coiled‐coils 1) and RIBC2 (RIB43A domain with coiled‐coils 2), which have similar domains. Previous studies revealed that RIBC1 is enriched in high‐fertile bull spermatozoa,^20^ and localized to the outer dynein arm of fish sperm axonemes.^21^ Similarly, the *RIBC2* gene is expressed in motile cilia in humans,^22^ and its coding protein co‐localizes with ciliary axonemes in human respiratory cells and the basal body in human retinal pigment epithelial cell lines.^23^ In addition, RIBC2 is located on the axonemes of cilia in *Xenopus* epithelial cells and is involved in ciliary beating.^24^, ^25^ Recently, cryo‐electron microscopy (cryo‐EM) and artificial intelligence (AI)‐based modeling showed that mouse RIBC1 and RIBC2 are MIPs on the A tubules of the DMTs of the sperm flagellum (Figure S1B).^11^, ^12^, ^26^ However, the function of RIBC1 and RIBC2 in mammalian reproduction and sperm flagella is unclear due to the lack of knockout (KO) animal models.

In this study, we generated *Ribc1* KO, *Ribc2* KO, and *Ribc1* and *Ribc2* double‐knockout (*Ribc1/2* DKO) mice to reveal the functions of the genes in vivo. We revealed that loss of RIBC1 impaired sperm velocity, while loss of RIBC2 did not affect sperm velocity. However, no structural abnormalities in the axonemal structure were observed in all KO sperm flagella by transmission electron microscopy (TEM).

## 2 MATERIALS AND METHODS

### 2.1 Animals

All mouse experiments were approved by the Institutional Animal Care and Use Committee of the University of Tokyo and the Animal Care and Use Committee of the Research Institute for Microbial Diseases, Osaka University. All animals were maintained under institutional guidelines. ICR and B6D2F1 mice were purchased from Japan SLC (Shizuoka, Japan) or CLEA Japan (Tokyo, Japan). In this study, we generated genetically modified mouse lines, *Ribc1* KO, *Ribc2* KO, and *Ribc1/2* DKO mice on a B6D2F1 background. KO mouse lines used in this study were deposited and available through either Riken BioResource Center (Riken BRC; Tsukuba, Japan) or the Center for Animal Resources and Development, Kumamoto University (CARD; Kumamoto, Japan). The *Ribc1* KO mouse line was deposited under the name B6D2‐*Ribc1^em1Osb^*, and the stock ID number is 11465 (Riken BRC) or 3109 (CARD), respectively. The *Ribc2* KO mouse line was deposited under the name B6D2‐*Ribc2^em1Osb^*, and the stock ID number is 11989 (Riken BRC) or 3358 (CARD), respectively.

### 2.2 In silico polymerase chain reaction (PCR)

The Mammalian Reproductive Genetics Database V2 (https://orit.research.bcm.edu/MRGDv2)^27^, ^28^, ^29^, ^30^ was used to examine the expression pattern of mouse and human mRNA in various organs and each cell type of the testis. The accession numbers in Ensembl for each gene are as follows: Human *RIBC1* (ENSG00000158423), Human *RIBC2* (ENSG00000128408), Mouse *Ribc1* (ENSMUSG00000025257), and Mouse *Ribc2* (ENSMUSG00000022431).

### 2.3 Generation of antibodies

A rabbit polyclonal antibody was produced by immunization with mouse RIBC1 polypeptide C plus RELAAIEARRNREKERQSR (anti‐RIBC1 Pos1) or C plus KGMSAEQRAAIRKTQETQR (anti‐RIBC1 Pos2) and RIBC2 polypeptide (C plus SQPTEDYFSQFN). We confirmed both antibodies detect specific signals for each protein (Figures S2D and S3D). The antibodies were purified from serum using the polypeptide and SulfoLink coupling resin (Thermo Fisher Scientific, Waltham, MA, USA). Antibodies against IZUMO1 (KS064‐125)^31^ and SLC2A3 (KS64‐10)^32^ used in this study were generated as previously described.

### 2.4 Sperm head–tail separation

Sperm head–tail separation was performed as previously described^33^. Sonication (amplitude 35%, 10 s, five times) was performed after the collection of spermatozoa from the cauda epididymis into phosphate‐buffered saline (PBS), Handy Sonic UR‐20P, Tomy Seiko, Tokyo, Japan). The supernatant was removed after centrifuging at 4°C, 15,000×*g*, 15 min. The precipitate was suspended in a 90% Percoll solution (Cytiva, Tokyo, Japan) in PBS. After centrifuging at 4°C,15,000×*g*,15 min, sperm tails were localized in the upper layer and the heads in the lower layer. Each fraction was suspended in 5 volumes of PBS and centrifuged at 4°C,10,000×*g*,15 min. The supernatant was removed, and the proteins were extracted with a protein lysis buffer containing 6 M urea, 2 M thiourea, and 2% sodium deoxycholate.

### 2.5 Fractionation of sperm protein with Triton X‐100 and sodium dodecyl sulfate lysis buffers

Fractionation of sperm protein was performed as previously described.^34^ Spermatozoa were suspended in 1% Triton X‐100 lysis buffer (50 mM NaCl, 20 mM Tris‐HCl, pH 7.5, protease inhibitor), incubated at 4°C for 2 h, and centrifuged at 15,000×*g* for 10 min. The insoluble fraction (precipitate) was suspended in 1% sodium dodecyl sulfate (SDS) lysis buffer (75 mM NaCl, 24 mM EDTA, pH 6.0) and incubated at room temperature for 1 h. The SDS insoluble fraction (precipitate) was dissolved in the sample buffer.

### 2.6 Western blot analysis

Protein extraction from testes was performed by homogenization with protein lysis buffer as previously described.^35^ Protein lysates were separated by SDS‐PAGE and transferred to a nitrocellulose membrane or a polyvinylidene difluoride membrane. The membrane was then blocked with 5% or 10% skim milk (Wako, Tokyo, Japan) in TBS‐T (0.05% Tween20 in tris‐buffered saline) for 1 h, and incubated overnight at 4°C with primary antibodies. The membranes were washed with TBS‐T and incubated with the horseradish peroxidase‐conjugated secondary antibodies for 1 h or 2 h at room temperature. After washing with TBS‐T, ImmunoStarLD (Wako), SuperSignal West Pico Chemiluminescent Substrate (Thermo Fisher Scientific), Chemi‐Lumi One Super (Nacalai Tesque, Kyoto, Japan), or Chemi‐Lumi One Ultra (Nacalai Tesque) were used as a chemiluminescence reagent and images were taken with the Amersham Imager 600 (Cytiva) or Amersham ImageQuant 800 (Cytiva). The antibodies used in this study and their dilution factors are listed in Table S2.

### 2.7 Generation of knockout mice by the CRISPR/Cas9 system

Two guide RNAs (gRNAs) were designed for *Ribc1* and *Ribc2*, respectively, using CRISPRdirect.^36^ Table S1 shows the gRNA sequences. Generation of *Ribc1* KO and *Ribc2* KO mouse lines was performed as previously described.^37^, ^38^ Ribonucleoprotein (RNP) complexes containing crRNA (Merck, Darmstadt, Germany), tracrRNA (Merck), and Cas9 (Thermo Fisher Scientific) were introduced into fertilized eggs obtained from B6D2F1 × B6D2F1 mating by electroporation using a NEPA21 Super Electroporator (Nepagene, Tokyo, Japan). The embryos obtained were implanted into the oviduct of pseudopregnant ICR mice. The mice born were crossed with wild‐type (WT) B6D2F1 mice, and heterozygous KO mice were bred with siblings to produce KO mouse lines. The *Ribc1/2* DKO mouse line was generated by crossing *Ribc*1 KO and *Ribc*2 KO mice. Genotyping of the mutant alleles was performed by genomic polymerase chain reaction (PCR) and Sanger sequencing. Protein expression was confirmed by Western blot analysis and found *Ribc1* KO testis does not express RIBC1 (Figure S2D) and *Ribc2* KO testis does not express RIBC2 (Figure S3D). These results indicate that both KO mouse lines were successfully generated. The gRNAs and the primers used in the present study are listed in Table S1.

### 2.8 Fertility analysis

Male fertility was evaluated by cohabiting one sexually mature male mouse with three sexually mature females for 2–3 months and examining the number of pups per mating. Mating was confirmed by checking for vaginal plugs in female mice every weekday morning.

### 2.9 Histological analysis of testis and cauda epididymis

Hematoxylin and periodic acid‐Schiff (He‐PAS) staining of testis sections was performed as previously described.^39^Testis was fixed overnight at 4°C with Bouin's fixative (Polysciences., Inc., Warrington, PA, USA), and embedded in a paraffin block. Five micrometer sections were sliced with a Microm HM325 microtome (Leica Biosystems, Nussloch, Germany), placed on glass slides, and treated with 1% periodic acid (Nacalai Tesque) and Schiff's reagent (Wako), followed by treatment with Mayer's hematoxylin solution (Wako). For hematoxylin and eosin (HE) staining of cauda epididymis, 5 µm sections were treated with Mayer's hematoxylin solution, followed by treatment with eosin Y solution (Wako). The sections were observed using a BX53 microscope (Olympus, Tokyo, Japan) or an Axio Imager M2 microscope (Carl Zeiss, Jena, Germany).

### 2.10 Analysis of sperm morphology and sperm motility

For observing sperm morphology, spermatozoa were collected from the cauda epididymis, placed on glass slides, and observed under a BX53 microscope (Olympus). Sperm motility analysis was performed as previously described.^40^ Spermatozoa were obtained from the cauda epididymis into Toyoda Yokoyama Hoshi (TYH) medium^41^ and incubated at 37°C, 5% CO_2_. After 10 or 120 min of incubation, sperm motility was analyzed using a CEROS II sperm analysis system (Hamilton Throne Biosciences, Beverly, MA). Sperm flagellar motility patterns were observed using a BX53 microscope (Olympus) with a high‐speed camera (HAS‐L1, Ditect, Tokyo, Japan). Flagellar waveform patterns were observed using the sperm motion analyzing software (BohBohSoft, Tokyo, Japan).

### 2.11 Transmission electron microscope analysis of sperm flagellum

After the cauda epididymis was fixed with 4% paraformaldehyde in PBS at 4°C, the sample preparation was performed as described previously.^33^, ^42^ The samples were observed using a JEM‐1400 plus electron microscope (JEOL, Tokyo, Japan) at 80 kV with a CCD Veleta 2K × 2K camera (Olympus).

### 2.12 Statistics

Statistical analysis was performed using GraphPad Prism 10 (GraphPad Software, San Diego, CA, USA) with one‐way ANOVA followed by Tukey's multiple comparisons test, and *P *< 0.05 was considered significant. Error bars in the graphs indicate mean ± standard deviation (SD).

## 3 RESULTS

### 3.1 Both *Ribc1* and *Ribc2* are expressed in the mouse sperm flagellum

**FIGURE 1:**
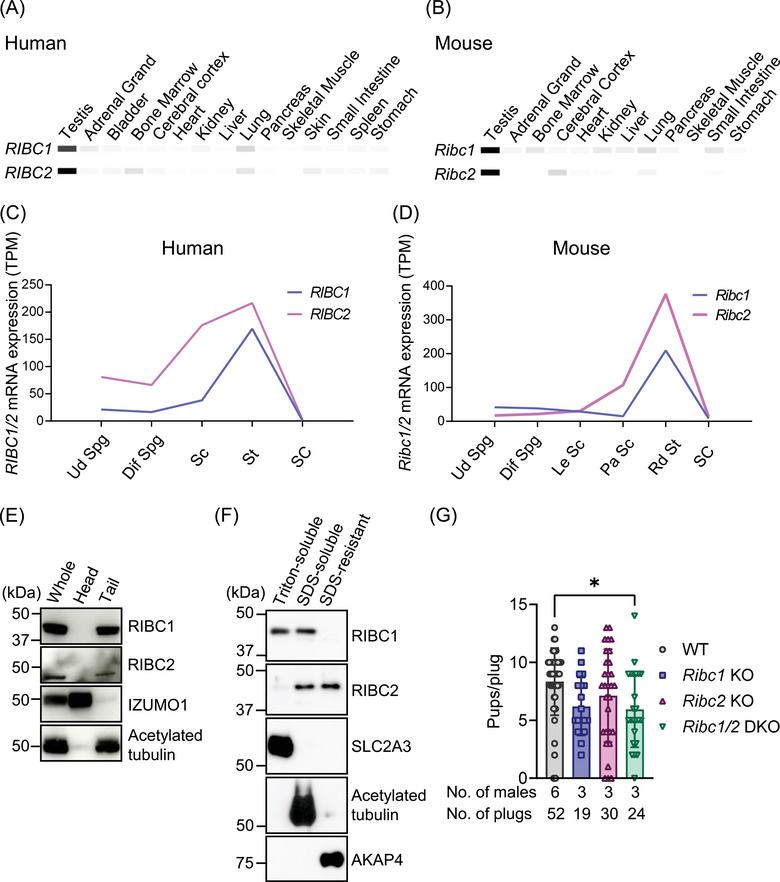
Both *Ribc1* and *Ribc2* are expressed in mouse sperm flagellum. Expression profiles of *RIBC1* and *RIBC2* in multiple human tissues from MRGDV2 RNA‐Seq datasets. The minimum TPM value was set to 0 and the maximum TPM value to 50. (A) Expression profiles of *Ribc1* and *Ribc2* in multiple mouse tissues from MRGDV2 RNA‐Seq datasets. The minimum TPM value was set to 0, and the maximum TPM value was set to 50. (B,C) Expression profiles of *RIBC1* and *RIBC2* in human testis from MRGDV2 RNA‐Seq datasets. Ud Spg, undifferentiated spermatogonia; Dif Spg, differentiated spermatogonia; Sc, Spermatocytes; St, Spermatids; SC, Sertoli cells. (D) Expression profiles of *Ribc1* and *Ribc2* in mouse testis from MRGDV2 RNA‐Seq datasets. Ud Spg, Undifferentiated spermatogonia; Dif Spg, differentiated spermatogonia; Le Sc, Leptotene spermatocytes; Pa Sc, Pachytene spermatocytes; Rd St, round spermatids; SC, Sertoli cells. (E) Western blot analysis using sperm head and tail fractions. Both RIBC1 and RIBC2 were detected in the tail fraction. IZUMO1 and acetylated tubulin were detected as markers for sperm heads and tails, respectively. (F) Western blot analysis using fractionated proteins of mouse spermatozoa. RIBC1 was found in the Triton‐soluble and SDS‐soluble fractions, whereas RIBC2 was found in the SDS‐soluble and SDS‐resistant fractions. SLC2A3, acetylated tubulin, and AKAP4 were used as markers for the Triton‐soluble, SDS‐soluble, and SDS‐resistant fractions, respectively. Number of litters born per plug detected. The average litter size was decreased in all KO mouse lines compared with WT. However, only *Ribc1/2* DKO male mice showed statistically significant differences with WT. *P *= 0.069 (WT vs. *Ribc1* KO), *P *= 0.359 (WT vs. *Ribc2* KO), and *P = 0.017 (WT vs. *Ribc1/2* DKO), one‐way ANOVA followed by Tukey's multiple comparison test (G). SDS, sodium dodecyl sulfate; KO, knockout; WT, wild type.

We investigated the tissue expression patterns of human *RIBC1* and *RIBC2*, and mouse *Ribc1* and *Ribc2* using the Mammalian Reproductive Genetics Database. In both humans and mice, *RIBC1* (*Ribc1*) and *RIBC2* (*Ribc2*) were highly expressed in the testis (Figure 1A,B). The expression of human *RIBC1* and *RIBC2* peaked in spermatids, and the expression of mouse *Ribc1* and *Ribc2* peaked in round spermatids during spermatogenesis (Figure 1C,D).

To confirm RIBC1 and RIBC2 localization in the spermatozoa, we performed Western blot analysis using sperm head and tail fractions. These results show that both RIBC1 and RIBC2 were localized to the sperm tail (Figure 1E). To further analyze the localization of RIBC1 and RIBC2 in the flagellum, we fractionated sperm proteins into a Triton X‐100 soluble fraction containing transmembrane and cytosolic proteins, an SDS‐soluble fraction containing axonemal proteins, and an SDS‐resistant fraction that includes outer dense fibers and fibrous sheath proteins.^34^, ^43^ RIBC1 was found in the Triton‐soluble and SDS‐soluble fractions, and RIBC2 was found in the SDS‐soluble and SDS‐resistant fractions (Figure 1F). RIBC1 and RIBC2 have been reported to be MIPs in the DMTs of the sperm flagellum,^11^, ^12^ so their presence in the SDS‐soluble fraction is reasonable. However, RIBC1 and RIBC2 were also detected in the Triton X‐100 soluble and SDS‐resistant fraction, respectively, which suggests that RIBC1 and RIBC2 could be localized elsewhere in addition to the DMTs.

### 3.2 Loss of both *Ribc1* and *Ribc2* impairs male fertility in mice

Since mouse *Ribc1* and *Ribc2* were abundantly expressed in the testis, and both are MIPs of the DMTs of the sperm flagellum^11^, ^12^, RIBC1 and RIBC2 may have an important role in sperm function. To check their functions in vivo, we generated *Ribc1* KO mice, *Ribc2* KO mice, and *Ribc1/2* DKO mice using the CRISPR/Cas9 system. Two gRNAs for generating *Ribc1* KO mice were designed to delete almost all the *Ribc1* open reading frames (ORFs) on chromosome X (Figure S2A). A 4799 bp deletion in the *Ribc1* gene was determined by Sanger sequencing (Figure S2B) and genomic PCR was used for genotyping mice (Figure S2C). Western blot analysis showed that RIBC1 was deleted in the testis of *Ribc1* KO mice (Figure S2D). Likewise, two gRNAs for generating *Ribc2* KO mice were designed to delete almost all the *Ribc2* ORFs on chromosome 15 (Figure S3A). A 9021 bp deletion in the *Ribc2* gene was determined by Sanger sequencing (Figure S3B), and the genotype was confirmed by genomic PCR (Figure S3C). Western blot analysis showed that RIBC2 was deleted in the testis of *Ribc2* KO mice (Figure S3D). We then generated *Ribc1/2* DKO mice by crossing *Ribc1* KO mice with mice carrying the *Ribc2* KO allele. To reveal the functions of RIBC1 and RIBC2 in male fertility, we mated individual single and double KO male mice with three WT female mice for 2–3 months. Mating results showed all KO male mice sired decreased average pup numbers per plug compared with WT (Figure 1G). However, only *Ribc1/2* DKO male mice showed statistically significant differences with WT (Figure 1G).

### 3.3 Spermatogenesis appears normal in *Ribc1* and *Ribc2* single KO and DKO mice

**FIGURE 2:**
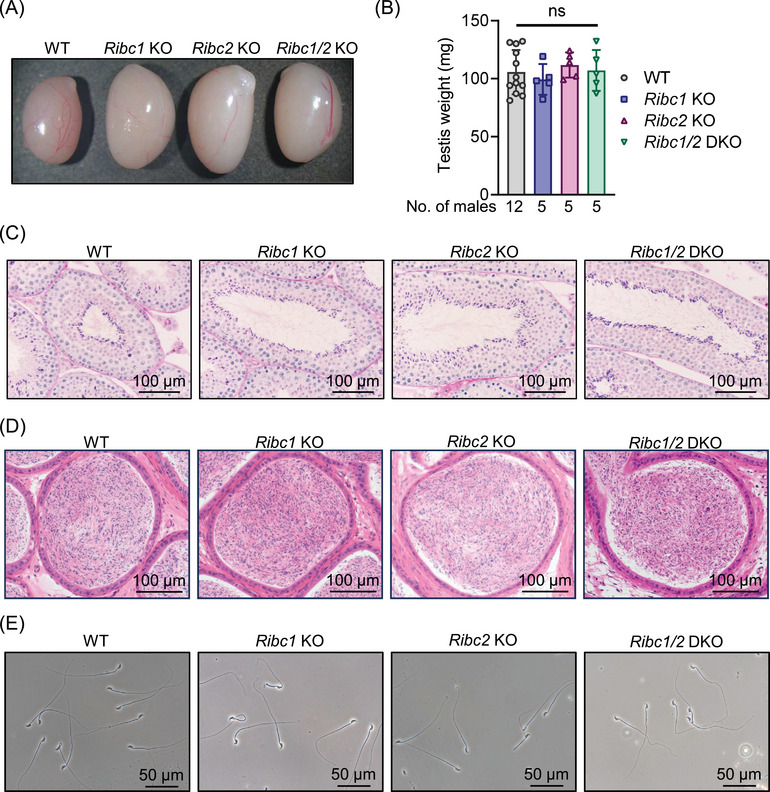
No obvious abnormalities are found in the testis and spermatozoa of *Ribc1* KO, *Ribc*2 KO, and *Ribc1*/2 DKO mice. (A) Gross morphology of testes in WT, *Ribc1* KO, *Ribc*2 KO, and *Ribc1*/2 DKO male mice. (B) Testis weight of WT, *Ribc1* KO, *Ribc*2 KO, and *Ribc1*/2 DKO male mice. No differences were observed (one‐way ANOVA followed by Tukey's multiple comparisons test, error bars represent SD). ns, not significant. (C) He‐PAS staining of testis sections in WT, *Ribc1* KO, *Ribc*2 KO, and *Ribc1*/2 DKO male mice. (D) HE staining of cauda epididymis in WT, *Ribc1* KO, *Ribc*2 KO, and *Ribc1*/2 DKO male mice. Sperm morphology from the cauda epididymis in WT, *Ribc1* KO, *Ribc*2 KO, and *Ribc1*/2 DKO male mice (E). KO, knockout; WT, wild type; SD, standard deviation.

To further investigate the effects of *Ribc1* and *Ribc2* deletions on spermatogenesis, we checked the testis of *Ribc1* KO, *Ribc2* KO, and *Ribc1/2* DKO male mice. No significant differences in gross morphology and weight were observed in all KO testis (Figure 2A,B). Histological analysis revealed no apparent differences in all KO spermatogenesis (Figure 2C). In addition, no differences were observed in the cauda epididymis between WT and all KO mice (Figure 2D). The morphologies of all KO spermatozoa were comparable to WT spermatozoa (Figure 2E).

### 3.4 Transmission electron microscopy revealed no structural abnormalities in sperm axonemes in the absence of RIBC1 and RIBC2

**FIGURE 3:**
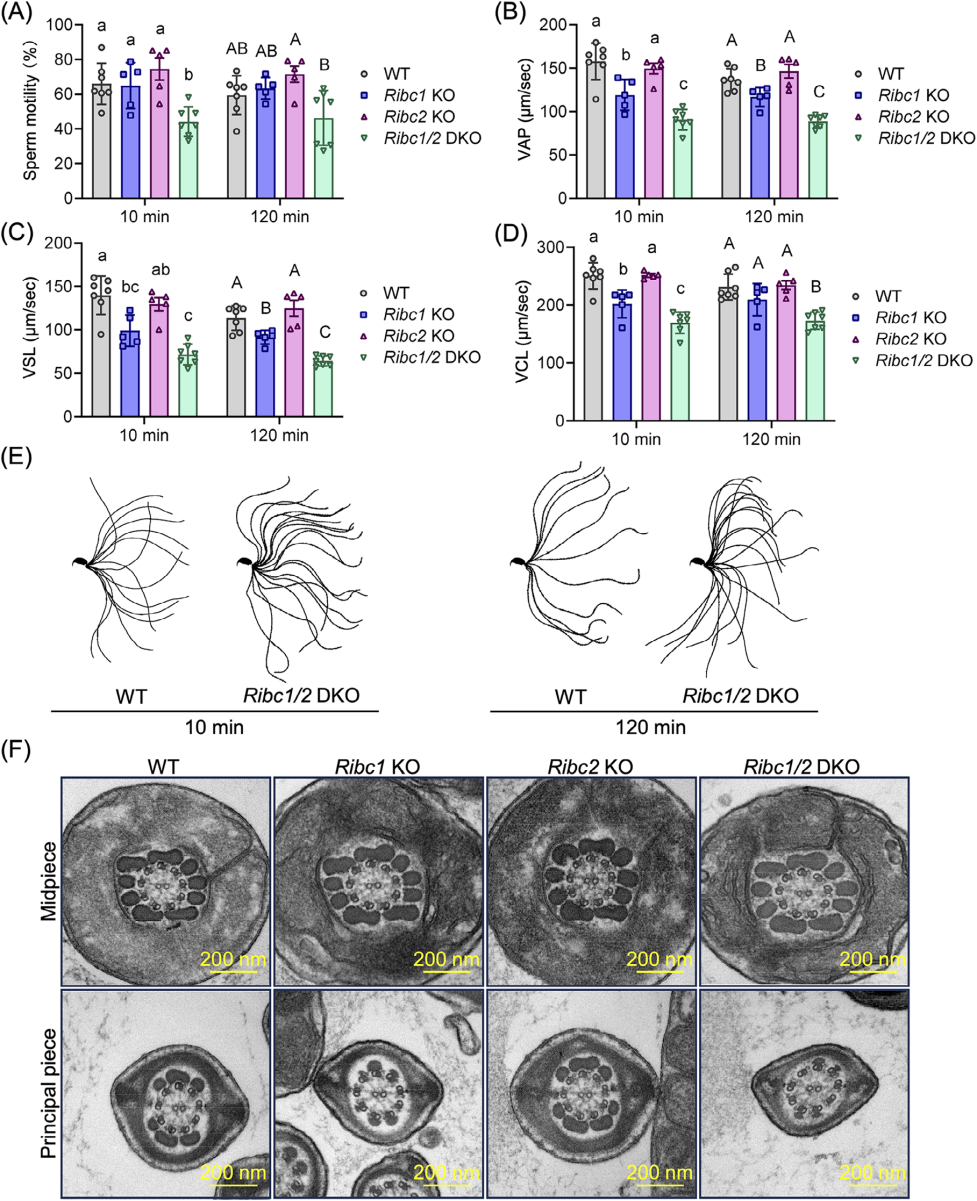
Sperm velocity is impaired in *Ribc1* KO and *Ribc1*/*2* DKO mice, but not in *Ribc2* KO mice. (A) Percentage of motile spermatozoa in WT, *Ribc1* KO, *Ribc*2 KO, and *Ribc1*/*2* DKO mice. Different letters (a vs. b, and A vs. B) indicate significant differences according to one‐way ANOVA followed by Tukey's multiple comparisons test (*P *< 0.05, error bars represent SD). (B) Average path velocity (VAP) of motile spermatozoa in WT, *Ribc1* KO, *Ribc*2 KO, and *Ribc1*/*2* DKO mice. Different letters (a vs. b, a vs. c, b vs. c, A vs. B, A vs. C, and B vs. C) indicate significant differences according to one‐way ANOVA followed by Tukey's multiple comparisons test (*P *< 0.05, error bars represent SD). (C) Straight‐line velocity (VSL) of motile spermatozoa in WT, *Ribc1* KO, *Ribc*2 KO, and *Ribc1*/*2* DKO mice. Different letters (a vs. bc, a vs. c, bc vs. ab, ab vs. c, A vs. B, A vs. C, and B vs. C) indicate significant differences according to one‐way ANOVA followed by Tukey's multiple comparisons test (*P *< 0.05, error bars represent SD). (D) Curvilinear velocity (VCL) of motile spermatozoa in WT, *Ribc1* KO, *Ribc*2 KO, and *Ribc1*/*2* DKO mice. Different letters (a vs. b, a vs. c, b vs. c, and A vs. B) indicate significant differences according to one‐way ANOVA followed by Tukey's multiple comparisons test (*P *< 0.05, error bars represent SD). (E) Flagellar waveform patterns of motile spermatozoa in WT, *Ribc1* KO, *Ribc*2 KO, and *Ribc1*/*2* DKO mice analyzed 10 or 120 min after incubation. The motility was videotaped at 200 frames/s. Single frames throughout one beating cycle were superimposed. Ultrastructure of sperm tails in WT, *Ribc1* KO, *Ribc*2 KO, and *Ribc1*/*2* DKO mice. Cross‐sections of the midpiece and the principal piece were observed using TEM (F). KO, knockout; WT, wild type; SD, standard deviation; TEM, transmission electron microscope.

We analyzed sperm motility using a computer‐assisted sperm analysis (CASA) system after 10 and 120 min of incubation in a capacitation medium. CASA revealed that although the percentage of motile spermatozoa was comparable between WT and *Ribc1* KO mice (Figure 3A), average path velocity (VAP), straight line velocity (VSL), and curvilinear velocity (VCL) after 10 and/or 120 min of incubation were significantly lower in *Ribc1* KO spermatozoa compared to WT (Figure 3B–D). On the other hand, all parameters of *Ribc2* KO mice were comparable to WT (Figure 3A–D). The percentages of motile spermatozoa, VAP, VSL, and VCL were significantly lower in *Ribc1/2* DKO spermatozoa compared to WT control mice except for that of motile spermatozoa at 120 min (Figure 3A–D). Furthermore, the percentages of motile spermatozoa at 10 min, VAP at 10 and 120 min, VSL at 120 min, and VCL at 10 and 120 min were significantly lower in *Ribc1/2* DKO spermatozoa compared to both *Ribc1* KO spermatozoa and *Ribc2* KO spermatozoa (Figure 3A–D). Throughout all KO spermatozoa, sperm motility and velocity did not change between 10 and 120 min of incubation (Figure 3A–D). We then analyzed the flagellar waveform patterns and found that the flagellar waveform pattern of *Ribc1/2* DKO was comparable to that of WT (Figure 3E). To investigate if the absence of RIBC1 and RIBC2 changes the structure of the axoneme, we used a TEM to observe axoneme structures. However, no obvious abnormalities in the structure of the sperm flagellum were observed in each KO spermatozoa (Figure 3F). Taken together, we revealed that loss of both RIBC1 and RIBC2 does not impair the axoneme structures of spermatozoa but impairs sperm velocity in mice.

## 4 DISCUSSION

Cryo‐EM and AI‐based modeling revealed that mouse RIBC1 and RIBC2 are MIPs of the DMTs of the sperm flagellum (Figure S1B).^11^, ^12^ In this study, we therefore generated KO mouse lines of both RIBC1 and RIBC2, and analyzed their functions in the sperm flagellum. The results showed that the loss of RIBC1 or RIBC2 alone did not affect male fertility (Figure 1G). Sperm motility analysis showed that *Ribc1* KO spermatozoa had reduced sperm velocity compared to WT (Figure 3B–D), indicating that RIBC1 has a role in sperm motility. On the other hand, *Ribc2* KO sperm velocities were comparable to WT mice. In human and mouse germ cells, *Ribc2* expression is higher than *Ribc1* expression (Figure 1C,D). This suggests that differences in expression levels of RIBC proteins cannot explain the effect on sperm motility. A possible explanation for the variation in sperm motility differences between *RIbc1* and *Ribc2* KO mouse lines may be related to the different locations of RIBC1 and RIBC2 on the luminal side of DMTs (Figure S1B). Our study also revealed that *Ribc1/2* DKO sperm velocities were significantly lower than those from *Ribc1* KO mice despite *Ribc2* KO sperm velocities being comparable with WT sperm (Figure 3A–D). These results suggest that the RIBC2 function depends on RIBC1 in sperm motility. We also found that the absence of RIBC1 and RIBC2 does not affect axoneme structure at the TEM level (Figure 3F). This result indicates that both RIBC1 and RIBC2 are not so pivotal for DMT structure integrity. Nevertheless, the reduced sperm motility (Figure 3A–D) suggests that TEM may not be suitable for detecting minor structural changes due to the absence of RIBC1 and RIBC2. Because neither RIBC1 nor RIBC2 have numerous contact proteins in the A tubule (Figure S1B), we hypothesize that their absence has a few effects on DMT structure. Minor structural abnormalities in *Ribc1/2 KO* spermatozoa may be detected by cryo‐EM analysis.

While RIBC1 and RIBC2 are assigned as MIPs of the sperm axonemes’ DMTs (Figure S1B),^11^, ^12^ each protein is also localized to a different fraction in the sperm tail (Figure 1F). This result suggests that both RIBC1 and RIBC2 localize elsewhere apart from DMTs or RIBC1 may detach from DMTs. However, because our antibodies against both RIBC1 and RIBC2 could not be used for immunofluorescent analysis, we could not determine where both proteins are located. Although the number of pups obtained decreased compared to WT mice, the DKOs were still fertile (Figure 1G). This result suggests that both RIBC1 and RIBC2 are not so significant for sperm functions in mice, despite their localization on DMTs. In *Chlamydomonas* and *Tetrahymena*, RIB43a, which has a structure similar to both RIBC1 and RIBC2, is predicted to be crucial for flagellar stability,^17^, ^18^ but both RIBC1 and RIBC2 in mice were not significant for flagellar stability (Figures 2E and 3F). This indicates that RIBC1 and RIBC2 functions in vertebrates may have changed from Rib43a in unicellular organisms.

This study revealed that the absence of RIBC1 causes a decrease in sperm velocity, but deletion of RIBC2 does not (Figure 3B–D), and the absence of both RIBC1 and RIBC2 causes a decrease in sperm motility (Figure 3A–D) and pup numbers (Figure 1G) compared with WT. A possible explanation for the reduced litter size in DKO mice is reduced sperm motility due to minor structural abnormalities caused by the loss of two RIBC proteins. Both RIBC1 and RIBC2 are MIPs in the A tubules of the DMTs, but their presence has few effects on sperm motility and axoneme structure. However, MIPs contribute to sperm motility and axoneme structure in mammals such as MNS1 and TEKTIP1.^15^, ^16^ Further analysis using KO mouse models would clarify other MIPs’ functions in sperm motility and axoneme structure in mammals.

## 5 CONCLUSIONS

This study performed a phenotypic analysis of *Ribc1* and *Ribc2* KO male mice. The results showed that the absence of RIBC1 causes a slight decrease in sperm velocity, but the deletion of RIBC2 does not. The absence of both RIBC1 and RIBC2 causes a decrease in sperm motility and pup numbers compared with WT. RIBC1 and RIBC2 are MIPs in the A tubules of the DMTs, but their absence does not affect the axonemal structure. However, minor structural abnormalities caused by the loss of two RIBC proteins might not be detected by TEM. We think reduced litter size in DKO mice is caused by reduced sperm motility due to minor structural abnormalities caused by the loss of two RIBC proteins.

## AUTHOR CONTRIBUTIONS

The study was designed by Kento Katsuma and Masahito Ikawa. Data were collected by all authors and analyzed and interpreted by Kento Katsuma and Masahito Ikawa. The manuscript was drafted by Kento Katsuma and Keisuke Shimada and revised by all authors. All authors read and approved the final version of the manuscript.

## CONFLICT OF INTEREST STATEMENT

The authors declare no conflicts of interest.

## Supporting information



Supporting information

## Data Availability

The data that support the findings of this study are available from the corresponding author upon reasonable request.
