# Supplementary material for: The absence of both RIBC1 and RIBC2 induces decreased sperm motility and litter size in male mice
Source: Andrology. 2025 Apr 23;14(2):545–54. doi: 10.1111/andr.70045 (PMC12842877; doi:10.1111/andr.70045)
Supplement: Supplementary file 1 — Supporting information [file ANDR-14-545-s001.pdf]

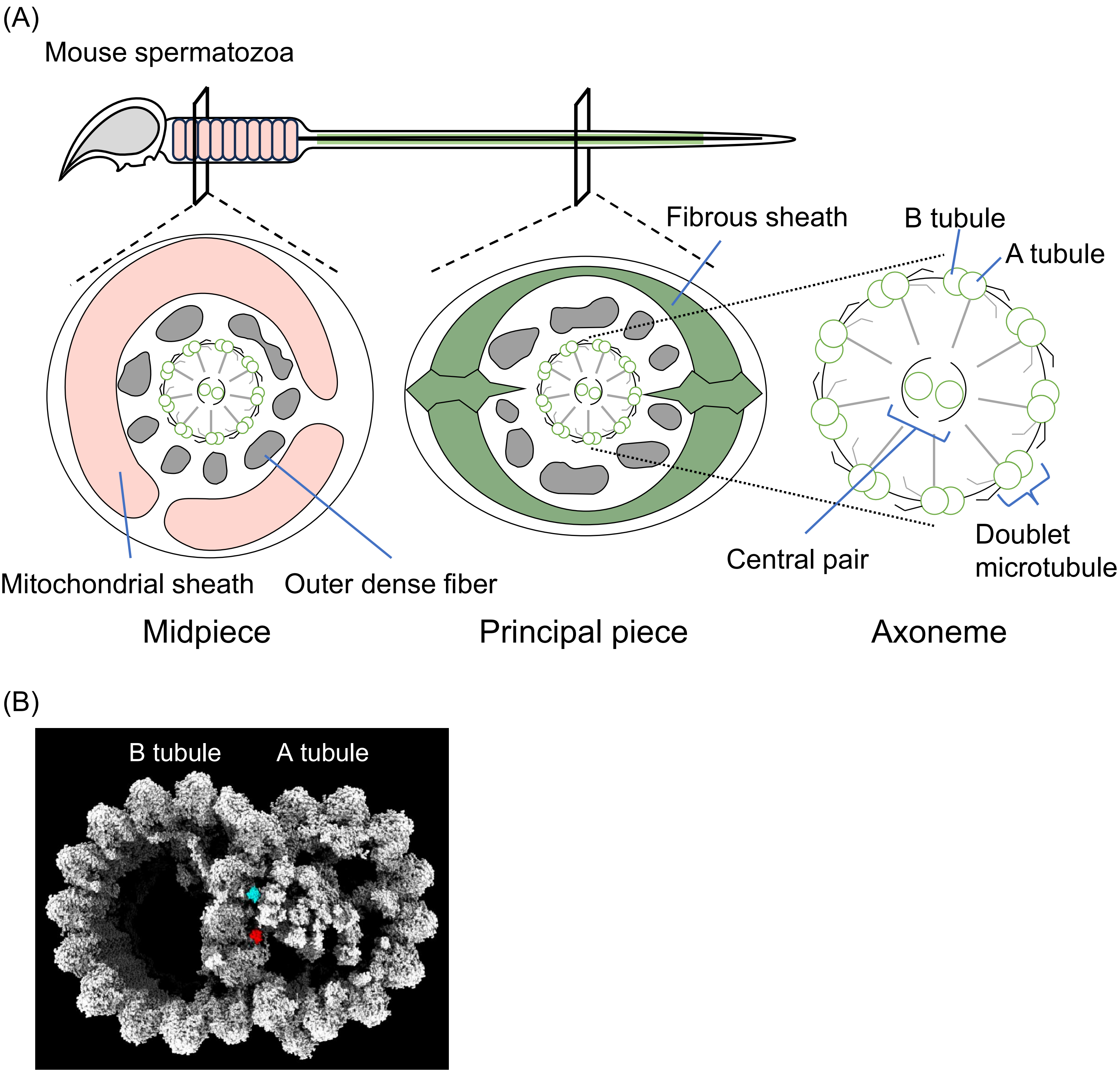

FIGURE S1. RIBC1 and RIBC2 are MIPs in the A tubules of the DMTs of the sperm flagellum
(A) Schematic of mouse mature spermatozoa including transverse sections of the midpiece and principal piece. Axoneme consists of a "9+2" microtubule structure with a central pair of singlet microtubules surrounded by nine peripheral microtubule doublets. (B) RIBC1 and RIBC2 are MIPs in the A tubules of the DMT of the mouse sperm flagella. This figure is generated in ChimeraX v1.8[26] using the cryo-EM structure of the DMT from mouse spermatozoa obtained from EMDB entry EMD-35823[11]. Cyan indicates RIBC1 and red indicates RIBC2, respectively.

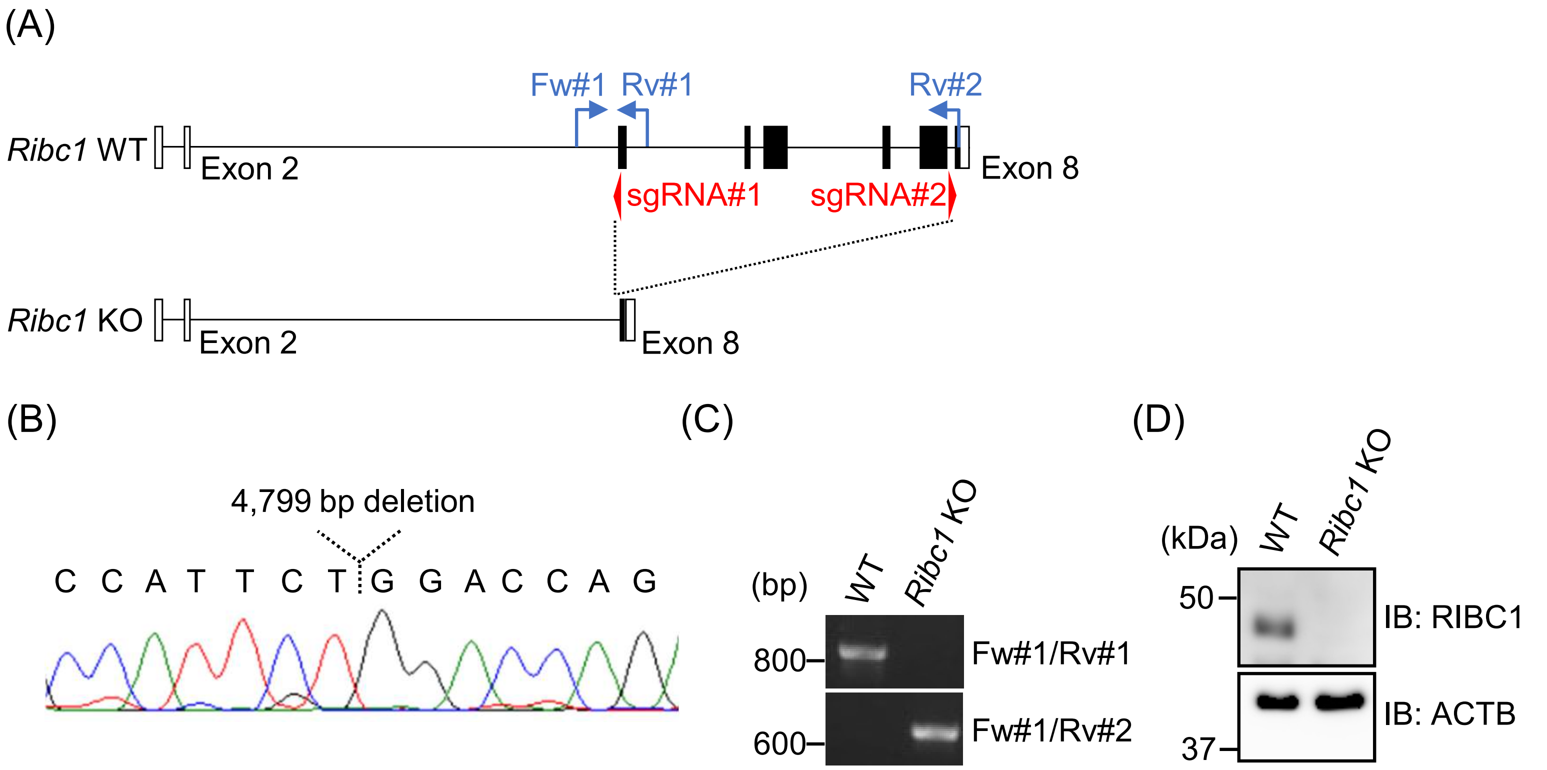

FIGURE S2. Generation of *Ribc1* KO mice

(A) KO strategy for generating *Ribc1* mutant mice. The direction of the red arrowheads indicates the direction of sgRNA (#1 and #2). Black boxes indicate coding sequences, and white boxes represent non-coding sequences. Fw#1, a forward primer for genotyping; Rv#1 and Rv#2, reverse primers for genotyping. (B) Genotyping of *Ribc1* mutant mice. Fw#1 and Rv#1 for detecting the WT allele, and Fw#1 and Rv#2 for detecting the KO allele. The primers illustrated in (A) were used, respectively. (C) Nucleotide sequence of the *Ribc1* KO allele confirmed by Sanger sequencing. (D) Protein expression of RIBC1 in the testis. The disruption of RIBC1 in *Ribc1* KO testis was confirmed. ACTB was used as loading control.

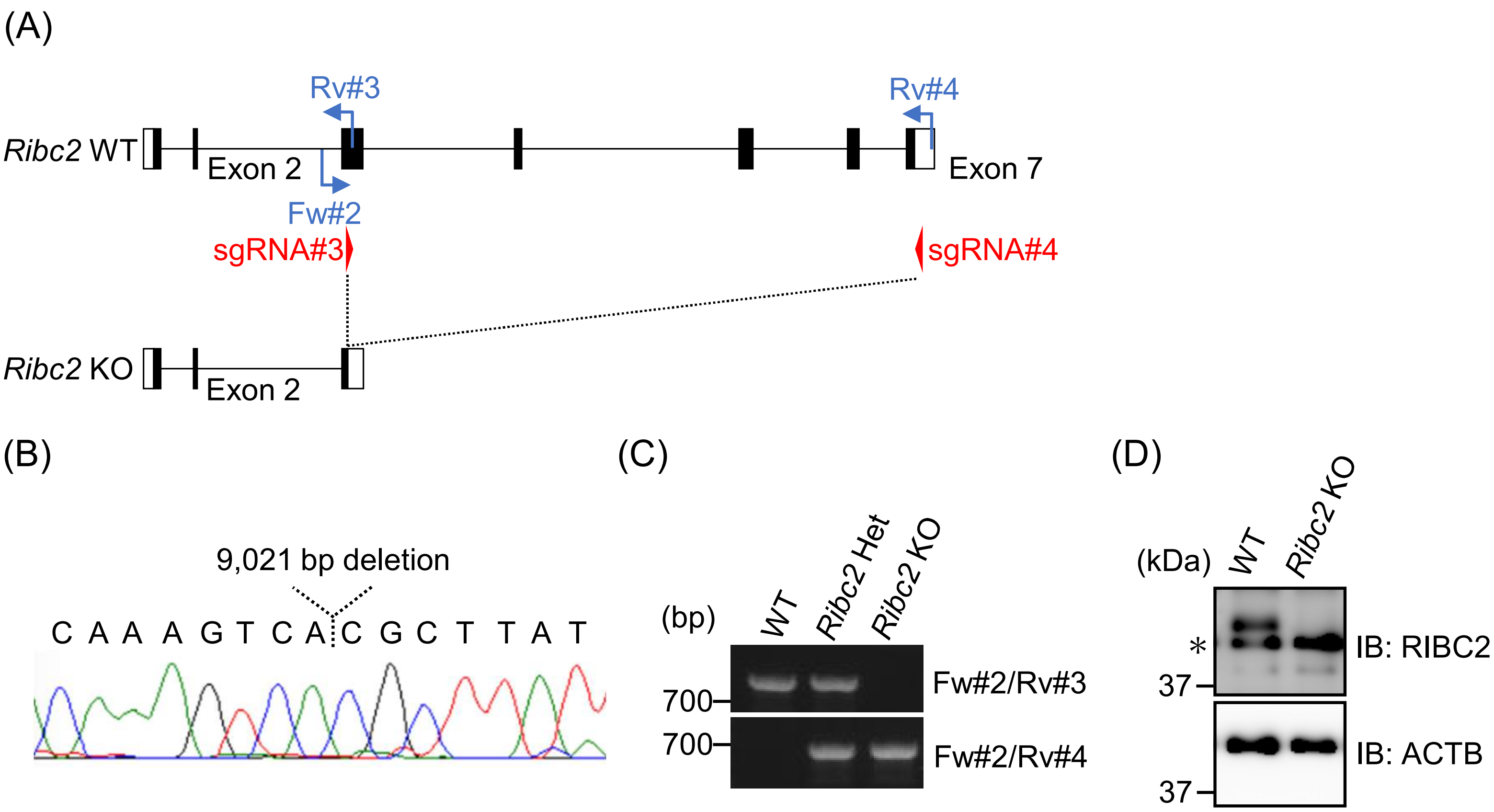

FIGURE S3. Generation of *Ribc*2 KO mice

(A) KO strategy for generating *Ribc2* mutant mice. The direction of the red arrowheads indicates the direction of sgRNA (#3 and #4). Black boxes indicate coding sequences, and white boxes represent non-coding sequences. Fw#2, a forward primer for genotyping; Rv#3 and Rv#4, reverse primers for genotyping. (B) Genotyping of *Ribc2* mutant mice. Fw#2 and Rv#3 for detecting the WT allele, and Fw#2 and Rv#4 for detecting the KO allele. The primers illustrated in (A) were used, respectively. (C) Nucleotide sequence of the *Ribc2* KO allele confirmed by Sanger sequencing. (D) Protein expression of RIBC2 in the testis. The disruption of RIBC2 in *Ribc2* KO testis was confirmed. ACTB was used as loading control. The asterisk indicates a nonspecific band.

Table S1.

Method	Symbol	Sequence (5'→3')
gRNA	*Ribc1* sgRNA#1	TATACATCTGGAGCAACGAG
	Ribc1 sgRNA#2	GGTAGGTGGGAGGAATGACG
	Ribc2 sgRNA#3	CATGTGCATGGCGCATGACC
	Ribc2 sgRNA#4	GAAATAAGCGTGACTCCTAG
Genotyping	*Ribc1* Fw#1	ACAGTCTGGCTCGAGCTTACAC
	Ribc1 Rv#1	GTGCAAGAACTGACCAGAGGAGAG
	Ribc1 Rv#2	CGCAGTCGGTTGATTGGTGTAGATG
	Ribc2 Fw#2	CAATCCCAGCCTCATTCCTCACATC
	Ribc2 Rv#3	GAAACTGGGTTAGAGGGTGACTTGG
	Ribc2 Rv#4	CTTTATGGTGGGTGAGAAGCTGAGG

Table S1. The gRNAs and primers used in this study

Table S2.

Antibody	Host	Clone No.	Company	Catalog No.	Lot No.	Dilution
anti-RIBC1 Pos1	Rabbit	Polyclonal	Merck	Custom	-	1:200 or 1:300
anti-RIBC1 Pos2	Rabbit	Polyclonal	Merck	Custom	-	1:200
anti-RIBC2	Rabbit	Polyclonal	Merck	Custom	-	1:200 or 1:300
anti-ACTB	Mouse	AC-15	Merck	A5441	055K4854	1:3000
anti-IZUMO1	Rat	KS64-125	In house[30]	Custom	-	1:1000
anti-acetylated tubulin	Mouse	6-11B-1	Merck	T7451	149704	1:5000 or 1:10000
anti-SLC2A3	Rat	KS64-10	In house[31]	Custom	-	1:1000
anti-AKAP82 (AKAP4)	Mouse	25/AKAP82	BD Biosciences	611564	9066953	1:1000

Table S2. Antibodies used for this study
